# LncRNA H19 promotes the differentiation of bovine skeletal muscle satellite cells by suppressing Sirt1/FoxO1

**DOI:** 10.1186/s11658-017-0040-6

**Published:** 2017-06-23

**Authors:** Xiaochun Xu, Shengyue Ji, Weili Li, Bao Yi, Hengxin Li, Hongfu Zhang, Wenping Ma

**Affiliations:** 1 College of Biology Sciences and Engineering, Beifang University of Nationalities, Yinchuan, Ningxia People’s Republic of China; 2 State Key Laboratory of Animal Nutrition, Institute of Animal Science, Chinese Academy of Agricultural Sciences, No. 2 Yuanmingyuan West Road, Beijing, 100193 People’s Republic of China

**Keywords:** H19 lncRNA, Myoblast differentiation, Sirt1, FoxO1

## Abstract

**Background:**

H19 is a well-characterized Long noncoding RNA (lncRNA) that has been proven to promote myoblast differentiation in humans and mice. However, its mechanism of action is still not fully interpreted.

**Methods:**

Using RT-qPCR, we examined H19 RNA levels in various tissues from 1-week, 1-month, 6-month and 36-month old male cattle (i.e., newborn, infant, young and adult). The protein and mRNA levels of MyoG, MyHC, Sirt1 and FoxO1 in the satellite and C_2_C_12_ cells with an H19 silencing or overexpression vector were respectively detected using western blot and real-time qPCR.

**Results:**

H19 was highly expressed in skeletal muscle at all the studied ages. High expression of H19 was required for the differentiation of bovine satellite cells. Knockdown of H19 caused a remarkable increase in the myoblast-inhibitory genes Sirt1/FoxO1, suggesting that H19 suppresses Sirt1/FoxO1 expression during myogenesis. Western blotting analysis of co-transfection of Sirt1 or FoxO1 expression vectors with pcDNA-H19 indicated that Sirt1/FoxO1 overexpression neutralized the promotion of myoblast differentiation through transfection of pcDNA-H19.

**Conclusion:**

H19 promoted the differentiation of bovine skeletal muscle satellite cells by suppressing Sirt1/FoxO1.

**Electronic supplementary material:**

The online version of this article (doi:10.1186/s11658-017-0040-6) contains supplementary material, which is available to authorized users.

## Introduction

Skeletal myogenesis is a complex process that depends on the modulation of a series of genes. Myogenic differentiation antigen (MyoD) and myogenic factor 5 (Myf5) enable the differentiation of myogenic progenitors into myoblasts, and then myogenin (MyoG or Myf4) promotes the myoblasts to differentiate into myotubes [1, 2]. Several other factors negatively regulate the differentiation process, including forkhead proteins (FoxO), which inhibit myogenic differentiation by stabilizing Notch/Hes binding. In the absence of FoxO1, MyoD expression is elevated and myogenic differentiation is enhanced [3].

Long noncoding RNAs (lncRNAs) have recently emerged as an important class of gene expression regulators. They are localized both in the nucleus and the cytosol, and play an important role in gene expression regulation at every stage of life [4, 5]. The contributions of lncRNAs have thus far mostly been investigated in cancer and neurological disorders, while the role of lncRNA in myogenesis is still poorly reported. Only a few lncRNAs were proven to be involved in myogenic differentiation. For instance, linc-MD1 is a muscle-specific intergenic lncRNA that acts as a sponge for miR-133 and miR-135, preventing their suppression of MAML1 and MEF2C and activating muscle-specific gene expression [6]. These discoveries illustrate that lncRNAs have greater potential that remain to be discovered and characterized.

H19 is a well-characterized lncRNA in mammalian cells. It is highly expressed in human and mouse developing embryos and adult skeletal muscles [7] and it is upregulated during myoblast differentiation and muscle regeneration [8]. H19 functions as a post-transcriptional suppressor or miRNA sponge for let-7, helping to regulate the Igf2 signaling pathway [9, 10]. It also encodes the conserved microRNAs miR-675-3p and -5p, which target Smad1, Smad5 and Cdc6, and thus promotes skeletal muscle differentiation and regeneration [8].

Muscle satellite cells are a heterogeneous population of committed myogenic progenitors and non-committed stem cells located beneath the basal lamina of skeletal muscle fibers [11, 12]. Described as quiescent myoblasts, they are crucial for the repair of muscle injury [13, 14]. The isolation, culture, and regulation of differentiation of muscle satellite cells are important methods for muscle biological research.

Here, we report that H19 was highly expressed in skeletal muscle in tissues from cattle of all postnatal ages. Knockdown of H19 seriously hindered the differentiation of skeletal muscle satellite cells. Gene expression analysis after H19 silencing or overexpression revealed that its promoting effect might be associated with blocking of the Sirt1/FoxO1 signaling pathway. Our findings revealed a novel pathway of H19 in myogenesis regulation.

## Materials and methods

### Ethics statement

All the experimental procedures involving cattle were approved by the Institute of Animal Science, Chinese Academy of Agricultural Sciences. The cattle were monitored in a stress-free environment where they were given food and water ad libitum in a humidity- and temperature-controlled room in the Experimental Farm of the Institute of Animal Science in Beijing.

### Samples

The heart, intestinal smooth muscle (smth-muscle), tibia skeletal muscle (sk-muscle), liver, spleen, kidney and lung were sampled from six examples each of 1-week (1w, representing newborns), 1-month (1 m, representing infants), 6-month (6 m, representing young animals) and 36-month old male cattle (36 m, representing well-developed adults). Embryo samples were obtained from three cows that were one month pregnant (E30), i.e., at a moment when the fetus has not yet formed. The samples for each age were mixed for further experimental analysis, and all procedures were performed in triplicate.

### Expression profile of H19 in various tissues from cattle of different ages

Total RNA was isolated from the various tissue and embryo samples. A TaKaRa Reverse Transcription Kit was used to obtain the first chain cDNA. Subsequent real-time qPCR reactions were carried out in a final volume of 25 μl, using SYBR Premix Ex Taq (TaKaRa), 0.4 mM of primer and 200 ng of cDNA template. Each individual sample was run in triplicate wells. The reactions were initially denatured at 95 °C for 3 min followed by 35 cycles of 95 °C for 15 s and 60 °C for 1 min. Amplicon quantification was performed using ABI 7300 software (Applied Biosystem). The primers used in the reactions were: H19 (F: 5′-TTC CCA GCC GCC ACT TC-3′, R: 5′-GAG CCG CTC CTG TGACCT ACT-3′), 18S RNA (F: 5′-GAGAAACGGCTACCACATCC-3′, R :5′-GCCAGACTTGCCCTCCA-3′), and GAPDH RNA (F: 5′-GCAAGTTCAACGGCACAG-3′, R: 5′-CGCCAGTAGACTCCACGACAT-3′). The change in transcript abundance of all tested genes was calculated using the 2^-ΔΔ^Ct method. All mRNA amounts were normalized to the GAPDH or 18S RNA control.

### Generation of constructs

The pLenti-H19, pLenti-NTC (the vector without the H19 gene), and pcDNA-H19 (the overexpression vector of H19 gene) interference plasmids were effectively constructed and identified by Dingguo Changsheng Biotechnology Co. LTD., according to the manufacturer’s instruction. The pcDNA-Sirt1 and pcDNA-FoxO1 were donated by Professor Shihuan Kuang (Purdue University).

The full-length bovine H19 gene was amplified with the sequence released in Genbank (Accession number: AC_000186.1) as the template. Primers applied were: F: 5′-CGG GGT ACC TGC GTG GGA GGG TGA AAG A-3′, R: 5′-CCC AAG CTT GGA GGG ACA CTA GGC AAG ATG G-3′. The product was cleaved and ligated into the corresponding sites of pcDNA-3.1 plasmid and verified via sequencing.

### Cell culture and transfection

Satellite cells were isolated from three 36-month old healthy adult cattle using the previously reported method [15]. C_2_C_12_ cells were bought from Baili Biotechnology Co., Ltd. The satellite and C_2_C_12_ cells were transfered in growth medium consisting of DMEM/F12 (Invitrogen) with 10% FBS, then incubated in a humidified incubator with an atmosphere of 95% air–5% CO_2_ at 37 °C.

When the cell confluence reached about 70%, 6 μg of pLenti-H19, pLenti-NTC (negative control) or pcDNA-H19 were transfected or co-transfected with expression vectors of pcDNA-Sirt1 or pcDNA-FoxO1 into the satellite cells and C_2_C_12_ cells with Lipofectamine 3000 (Invitrogen) according to the manufacturer’s instructions. The cells were then induced to differentiate. After transfection for 96 h, the expressions of MyoG, MyHC, Sirt1 and FoxO1 were detected using western blotting and RT-qPCR.

### Differentiation assay

To induce myoblast differentiation, the growth medium was replaced with differentiation medium (DMEM supplemented with 2% horse serum) when cell confluence reached 80%. Thereafter, the cultured cells were fixed with 3.7% formaldehyde, and then microscopically analyzed to determine the fraction of MHC-positive cells and the fusion indices. Permeabilization and blocking of non-specific binding were performed using phosphate buffer solution (PBS, pH 7.4) containing 0.1% Triton X-100 and 0.2% bovine serum albumin (BSA) for 1 h. The cells were incubated with anti-MHC (1:50) for 1 h at room temperature, then washed three times with PBS containing 0.1% Triton X-100. Next, they were incubated with secondary antibodies for 45 min at room temperature. After subsequent incubation of the cells with Hoechst 33342 for 10 min, they were washed three times, and Fluoromount G staining was applied. Fluorescence detection was performed using an Olympus IX51 inverted microscope. Random fields were analyzed with a 20X objective. The cell differentiation ratio was analyzed with Cellometer Cell Counters and Cell Analysis Systems.

### RNA extraction and real-time qPCR

Total RNA was isolated from the satellite cells and C_2_C_12_ at the start and on each of the first 5 days of the differentiation induction. RT-PCR for H19 lncRNA was performed using TRIzol reagent (Invitrogen) following the manufacturer’s instructions. Amplicon quantification was performed using a real-time qPCR protocol that was in accordance with the protocol for expression level detection of H19 in various tissues (mentioned above). The primers used in the reactions were: H19 (F: 5′-TTC CCA GCC GCC ACT TC-3′, R: 5′-GAG CCG CTC CTG TGACCT ACT-3′); MyoG (F: 5′-GAG GAA GTC TGT GTC GGT GG-3′, R: 5′-CCA CGA TGG ACG TAA GGG AG-3′); MyoHC (F: 5′-GCA TCC CTA AAG GCA GGC TC-3′, R: 5′-GCC ACT TGT AGG GGT TGA CA-3′); Sirt1 (F: 5′-AGA ACC ACC AAA GCG GAA A-3′, R: 5′-TCC CAC AGG AGA CAG AAA CC-3′); Foxo1 (F: 5′-CCC AGG CCG GAG TTT AAC C-3′, R: 5′-GTT GCT CAT AAA GTC GGT GCT-3′); GAPDH (F: 5′-GCA AGT TCA ACG GCA CAG-3′, R: 5′-CGC CAG TAG ACT CCA CGA CAT-3′). The change in transcript abundance of all tested genes was calculated using the 2^-ΔΔ^Ct method. All mRNA amounts were normalized to the GAPDH control.

### Western blotting

25 μg of protein isolated from each sample were separated using 12% SDS-PAGE and electro-transferred to a PVDF membrane (Millipore) for immunoblot analysis. The following primary antibodies were used: anti-Sirt1 (Abcam, ab32441, 1:500), anti-FoxO1 (Abcam, ab39670, 1:500), anti-MyoG (Abcam, ab1835, 1:500), anti-MyHC (Abcam, ab15, 1:500) and anti-GAPDH (Santa Cruz, sc-166574, 1:800), which was used as the reference. After incubation with the appropriate HRP-conjugate secondary antibody, proteins were detected using a ChemiDoc XRS imaging system and analyzed using Quantity One software (Bio-Rad). The protein abundance was normalized to the GAPDH gene. pLenti-NTC was used as a negative control. The blank control was proteins of the satellite and C_2_C_12_ cells without any treatment, but cultivated for the same period. GAPDH was used as an internal control.

### Statistical analysis

All data were presented as means ± S.E.M. of three biological replicates. Two-tailed Student’s *t*-test was employed to determine p values. The significance was set at *p* < 0.05 (*n* = 3).

## Results

### H19 was highly expressed in skeletal muscle in cattle of all ages

**Fig. 1:**
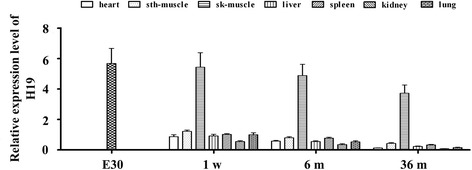
The expression profiles of H19 in various tissues from male cattle of different postnatal stages. GAPDH RNA is the reference gene. The relative expression in satellite and C_2_C_12_ cells during differentiation was calculated according to the 2^-ΔΔCt^ method. sth-muscle: smooth muscle; sk-muscle: tibia skeletal muscle; 1w: six 1-week old cattle, 1 m: 1-month old cattle, 6 m: 6-month old cattle, 36 m: 36-month old cattle. E30: samples from embryos after one month of development. The error bars were calculated with three repetitions

H19 RNA levels in various tissues of 1-week, 1-month, 6-month, and 36-month old male cattle were determined. The embryo was regarded as a positive control for H19, based on its high expression level at the embryo stage. To verify the accuracy of the results, GAPDH RNA (Fig. 1) and 18S RNA (Additional file 1: Figure S1) were used as reference genes. The results showed that the change patterns for H19 with 18S RNA and GAPDH RNA as reference genes were the same, indicating the high accuracy of the selected reference genes. Of the tissues we assessed, H19 was only highly expressed in skeletal muscle (sk-muscle) at all the ages. With age, H19 exhibited a moderate downward trend (Fig. 1, Additional file 1: Figure S1).

### H19 level rose during myoblast differentiation

**Fig. 2:**
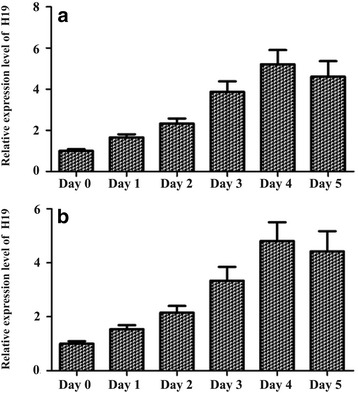
Expression levels of H19 during differentiation of bovine tibia skeletal muscle (sk-muscle) satellite cells (**a**) and C_2_C_12_ myoblast cells (**b**)

Satellite cells were obtained from skeletal muscle 36-month old cattle. When 80% confluence was reached, the cells, including the C_2_C_12_ cells, were induced to differentiate through the addition of 2% horse serum in DMEM (Invitrogen). The expression levels of H19 during myoblast differentiation were determined via RT-qPCR. The results showed that the expression level of H19 RNA in bovine skeletal muscle satellite cells and C_2_C_12_ myoblasts increased with differentiation time (Fig. 2a and b).

### Silencing H19 suppressed myoblast differentiation of skeletal muscle satellite cells

**Fig. 3:**
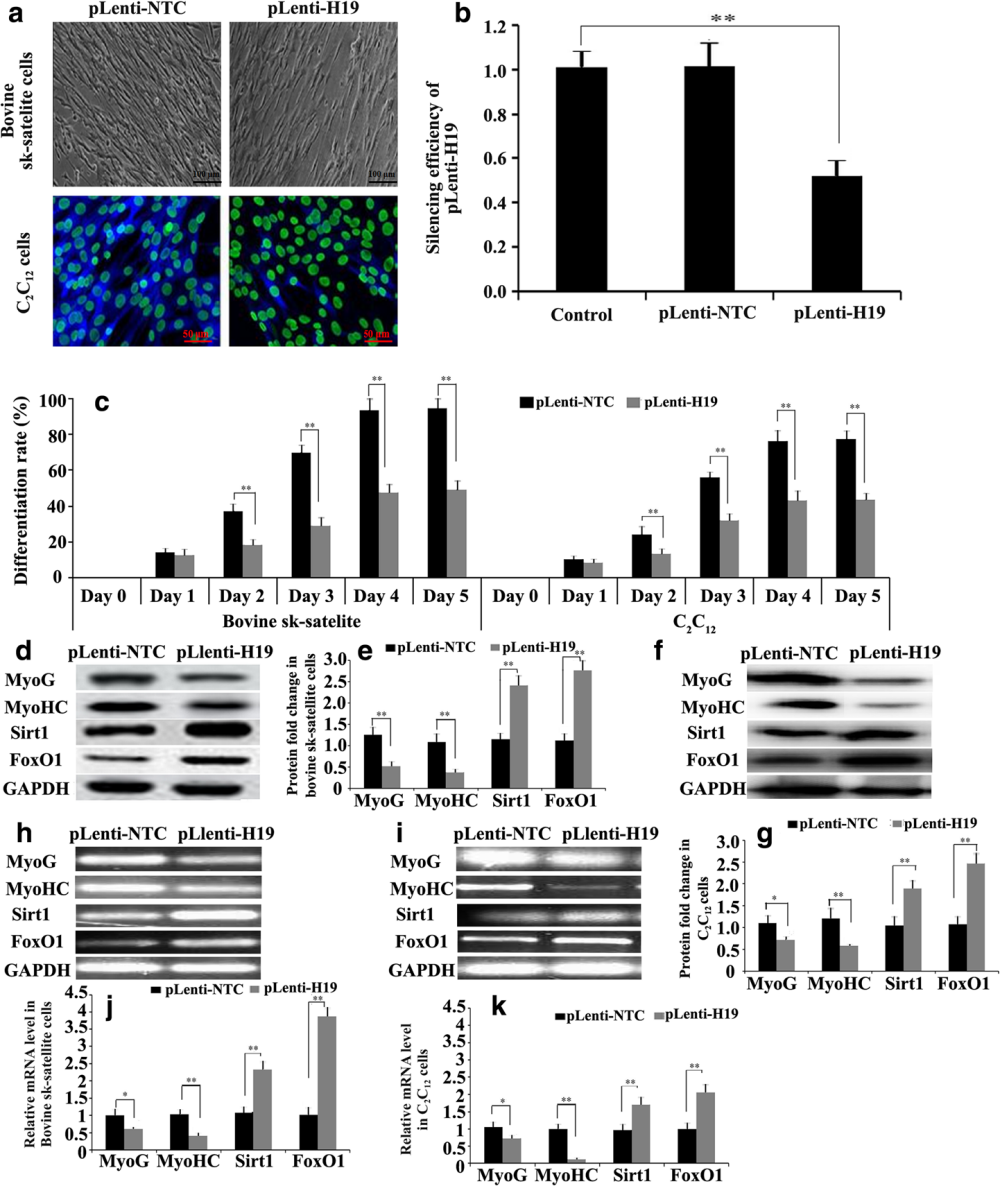
Knockdown of H19 suppressed the differentiation of bovine tibia skeletal muscle (sk-muscle) satellite and C_2_C_12_ cells. **a** The immunofluorescence results for C_2_C_12_ cells and satellite cells on day 4 of differentiation. **b** The silencing efficiency of pLenti-H19. Cells with pLenti-NTC were the negative control and wild-type cells were the blank control (control). **c** The differentiation rate of C_2_C_12_ cells and the satellite cells on days 1, 2, 3, 4 and 5. (**d**, **e**, **f** and **g**). Detection of myoblast marker and myoblast inhibitory genes based on the protein level after H19 was silenced by pLenti-H19 vector transfection in bovine sk-muscle satellite cells (**d**, **e**) and C_2_C_12_ cells (**f**, **g**). **h**, **i** RT-PCR results for bovine sk-muscle satellite cells (**h**) and C_2_C_12_ cells (**i**) after H19 was silenced by pLenti-H19 vector transfection. **j**, **k**. mRNA level of bovine sk-muscle satellite cells (**j**) and C_2_C_12_ cells (**k**) after H19 was silenced by pLenti-H19 vector transfection. pLenti-NTC was the negative control. The blank control was the mRNA of satellite and C_2_C_12_ cells without any treatment, with cultivation for the same number of days. GAPDH was internal control. *p < 0.05, **p < 0.01

To explore the effect of H19 on myoblast differentiation, a pLenti-H19 interference vector was transfected into the satellite and C_2_C_12_ cells, and then the cells were induced to differentiate. After 4 days, the cells were observed with a microscope and their differentiation ratio was analyzed. The fluoromount G staining and silencing efficiency results showed that the H19 gene was silenced after transfection of pLenti-H19 (Fig. 3a and b). The differentiation ratios of bovine satellite and C_2_C_12_ cells were both decreased by silencing H19 (Fig. 3c). Consistent with this change, the protein expression level of the myoblast differentiation maker genes *MyoG* and *MyHC* were significantly downregulated in bovine satellite cells (Fig. 3 d–g). Interestingly, we found that Sirt1 and FoxO1 expressions were remarkably upregulated (Fig. 3c). These were previously reported as myoblast inhibitors. As per the protein level change pattern, mRNA levels of *MyoG* and *MyoHC* in pLenti-NTC infected bovine sk-satellite and C_2_C_12_ cells were significantly lower than in pLenti-H19 infected cells on day 4 after the induction of differentiation, while the mRNA levels of *Sirt1* and *FoxO1* had the opposite trend (Fig. 3 h–k). These data suggest that high levels of H19 are required in bovine myoblast differentiation and that its function might be achieved through Sirt1 and/or FoxO1 suppression.

### Overexpression of Sirt1 and FoxO1 neutralized the promotion of myoblast differentiation by H19 overexpression

**Fig. 4:**
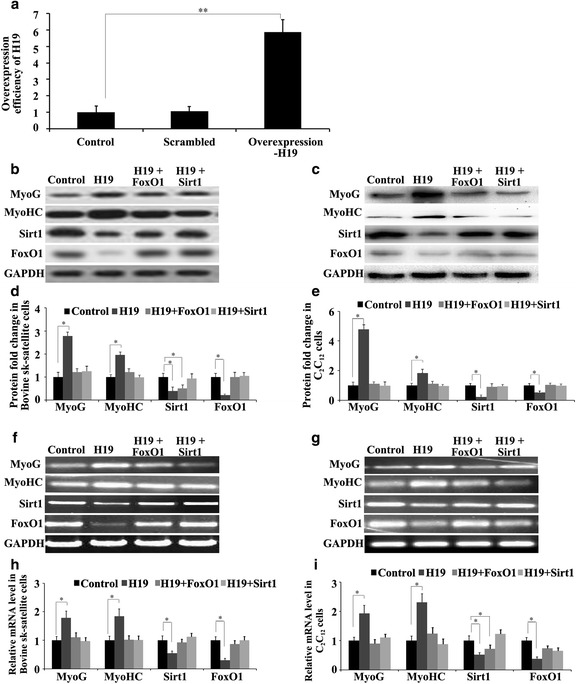
Sirt1/FoxO1 overexpression neutralized myoblast inhibition by H19. **a** The overexpression efficiency of H19. Cells without H19 transfection (scrambled) were the negative control and wild-type cells were the blank control (control). The protein levels of MyoG, MyHC, Sirt1 and FoxO1 in bovine tibia skeletal muscle (sk-muscle) satellite cells (**b** and **d**) and C_2_C_12_ cells (**c** and **e**) with H19 overexpression. **f**, **g** RT-PCR results for bovine sk-muscle satellite cells (**f**) and C_2_C_12_ cells (**g**) with H19 overexpression. **h**, **i** Real-time qPCR results for bovine sk-muscle satellite cells (**h**) and C_2_C_12_ cells (**i**) with H19 overexpression. The cells transfected with the vector without H19 (scrambled) were the control and GAPDH was the internal control. The protein and mRNA abundance was normalized to the GAPDH gene, *n* = 3, *p < 0.05, **p < 0.01

To verify the role of H19 in promoting myoblast differentiation through the suppression of Sirt1 and/or FoxO1, Sirt1 or FoxO1 expression vectors were co-transfected with pcDNA-H19 to the satellite cells and C_2_C_12_ cells. H19 was more highly expressed after pcDNA-H19 transfection (Fig. 4a). Western blotting and RT-qPCR analysis revealed that the expression levels of MyoG and MyoHC increased, while Sirt1 and FoxO1 expression decreased in the satellite cells and C_2_C_12_ cells with pcDNA-H19 transfection. After co-transfection with pcDNA-Sirt1 or pcDNA-FoxO1, the expression levels of MyoG and MyoHC decreased, while those for Sirt1 and FoxO1 increased (Fig. 4b–i), implying that Sirt1 and/or FoxO1 neutralized the promotion of MyoG and MyHC by overexpression of H19.

### Discussions

Here, the role of lncRNA H19 in myoblast differentiation was investigated in bovine skeletal muscle satellite cells. We found that H19 was more highly expressed in postnatal bovine skeletal muscle and revealed a novel mechanism of H19 in the promotion of myoblast differentiation. This mechanism may be associated with blocking of the Sirt1/FoxO1 signaling pathway.

H19 was one of the earliest identified lncRNAs [16]. It is thus far known that it plays roles in multiple biological processes, including negative regulation of body weight and cell proliferation [17], and it may also be involved in the formations of many types of cancer [7, 18–20].

The roles and underlying mechanisms of H19 in the regulation of myogenesis or regeneration have recently been deeply investigated. H19 was shown to promote skeletal muscle differentiation through the Igf2 signaling pathway or miR-675-mediated gene suppression [8, 9]. We found that the differentiation of bovine skeletal muscle satellite cells and C_2_C_12_ cells were suppressed (Fig. 3b) and that the expressions of Sirt1 and FoxO1 were enhanced after the silencing of H19 (Fig. 3d–k) but suppressed by its overexpression (Fig. 4). It was speculated that H19 promoted myoblast differentiation by suppressing the Sirt1/FoxO1 signaling pathway. Further study is needed to investigate the underlying mechanism.

Based on their site of action and the level of gene expression, lncRNAs can be divided into functional categories, but since some lncRNAs plays distinct roles in multiple processes or the same role in dissimilar pathways, this distinction is not always easy or adequate. As an example, H19 has been verified to function positively in some cancers but negatively in other cancers [21–23], and in myoblast differentiation, H19 exhibits several mechanisms for the same promoting effect.

In mammals, the majority of the transcriptional output is noncoding [24]. Compared to the well-studied coding genes, lncRNAs undoubtedly have greater research potential, especially in myogenesis.

## Conclusion

H19 is required in the differentiation of bovine skeletal muscle satellite cells. its promoting effect might be associated with the blocking of the Sirt1/FoxO1 signaling pathway. Our findings revealed a novel pathway for H19 in the regulation of myogenesis.

## Additional file

Additional file 1: Figure S1. The expression profiles of H19 in various tissues of cattle at different postnatal stages. The reference genes is 18S RNA. The relative expression levels of H19 in satellite and C_2_C_12_ cells during differentiation were calculated according to the method of 2^-ΔΔCt^. (TIF 498 kb)

## Abbreviations

BSA: Bovine serum albumin
FoxO: Forkhead proteins
lncRNAs: Long noncoding RNAs
Myf5: Myogenic factor 5
MyoD: Myogenic differentiation antigen
PBS: Phosphate buffer solution
sk-muscle: Skeletal muscle
sth-muscle: Smooth muscle
