# Supplementary figures and images for: LncRNA H19 promotes the differentiation of bovine skeletal muscle satellite cells by suppressing Sirt1/FoxO1
Source: Cell Mol Biol Lett. 2017 Jun 23;22:10. doi: 10.1186/s11658-017-0040-6 (PMC5481879; doi:10.1186/s11658-017-0040-6)

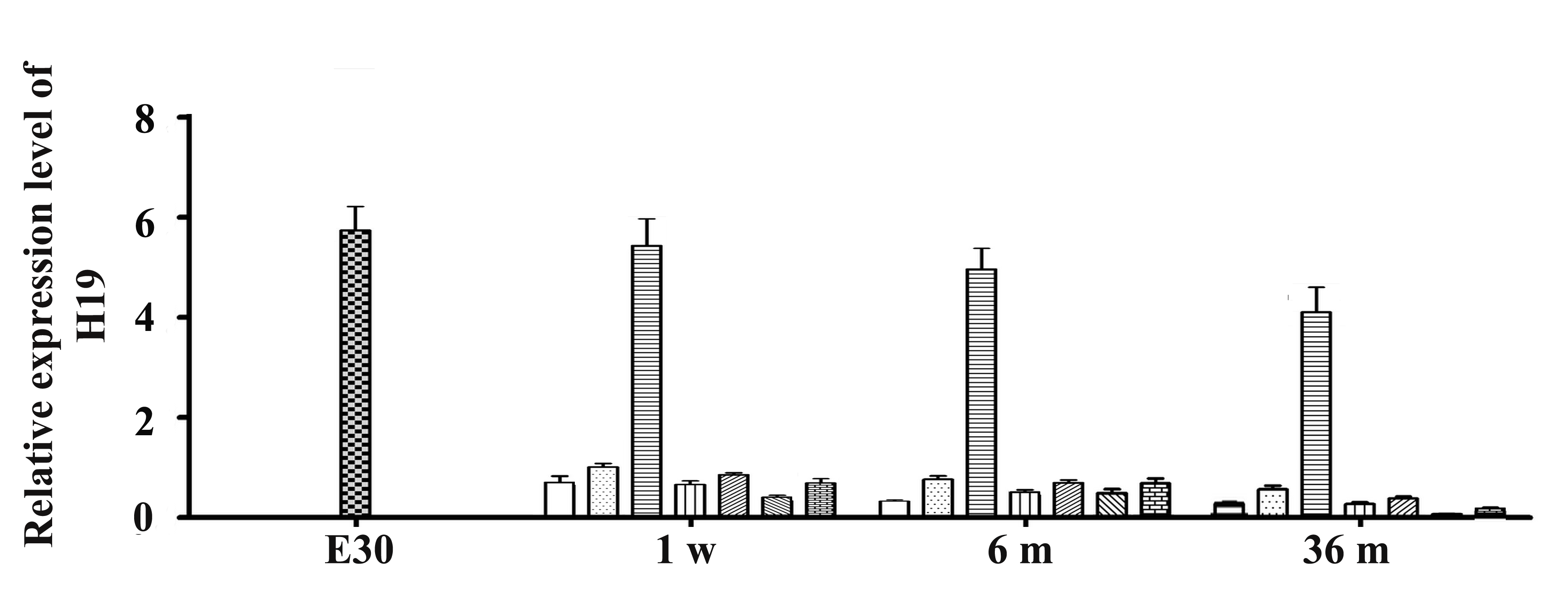

Supplement: Additional file 1: Figure S1. — The expression profiles of H19 in various tissues of cattle at different postnatal stages. The reference genes is 18S RNA. The relative expression levels of H19 in satellite and C2C12 cells during differentiation were calculated according to the method of 2-ΔΔCt. (TIF 498 kb) [file 11658_2017_40_MOESM1_ESM.tif]
